# Effects of chronic alcohol consumption on gene expression in the rat left ventricle: a transcriptomic analysis

**DOI:** 10.3389/fcvm.2026.1977478

**Published:** 2026-10-14

**Authors:** Weilun Ai, Carol A. Casey, Karuna Rasineni, Viswanathan Saraswathi

**Affiliations:** 1 Department of Internal Medicine, Division of Diabetes, Endocrinology, and Metabolism, University of Nebraska Medical Center, Omaha, NE, United States; 2 VA Nebraska-Western Iowa Healthcare System, Omaha, NE, United States; 3 Department of Internal Medicine, Division of Gastroenterology and Hepatology, University of Nebraska Medical Center, Omaha, NE, United States; 4 Department of Biochemistry and Molecular Biology, University of Nebraska Medical Center, Omaha, NE, United States

**Keywords:** alcohol, cardiomyopathy, circadian rhythm, interferon, RNA-Seq

## Abstract

**Objective:**

Chronic alcohol consumption is one of the leading causes of dilated cardiomyopathy. Alcohol-associated cardiomyopathy (ACM) is characterized by impaired cardiac function and ventricular dilation in individuals with a history of long-term alcohol consumption. Alcohol exerts adverse effects on the myocardium via mitochondrial dysfunction and chronic inflammation. However, the broader transcriptional changes associated with chronic alcohol exposure remain poorly understood.

**Methods:**

A chronic ethanol feeding model was utilized, in which male Wistar rats were fed a Lieber-Decarli ethanol diet containing 6.7% (v/v) ethanol (ET) or an isocaloric control (CONT) diet for 6 weeks. Left ventricular tissues were collected from ET-fed and CONT-fed rats. Differential gene expression was assessed using bulk RNA sequencing, followed by Ingenuity Pathway Analysis (IPA).

**Results:**

Transcriptomic analysis identified 194 upregulated and 199 downregulated genes in the left ventricles of ET-fed rats compared with CONT-fed rats. IPA revealed downregulated pathways related to the circadian clock and interferon signaling in the myocardium of ET-fed rats. In particular, the differentially altered genes included regulators of the circadian clock (*Bmal1, Npas2, Per1*, *Per2*) and interferon signaling (*Ifit2*, *Ifit3*, *Irf6*, *Irf9*). In contrast, pathways associated with mitochondrial RNA degradation, sheddase signaling, and neutrophil extracellular traps were upregulated in the myocardium following chronic ethanol exposure.

**Conclusion:**

Chronic ethanol exposure is associated with dysregulation of circadian rhythm and immune-related signaling pathways in the rat myocardium. These findings highlight molecular pathways that may contribute to the development of ACM and warrant further mechanistic investigation.

## 1 Introduction

Chronic heavy alcohol consumption is an important cause of dilated cardiomyopathy and can lead to alcohol-associated cardiomyopathy (ACM), which accounts for approximately 10% of dilated cardiomyopathy (1). ACM is characterized by ventricular dilation and impaired cardiac function (2). Chronic alcohol exposure promotes myocardial hypertrophy and dysfunction through multiple mechanisms, including inflammation, nitrative/oxidative stress, and increased collagen deposition in rats (3, 4). However, the molecular pathways linking chronic alcohol consumption to myocardial injury and cardiac dysfunction remain poorly understood.

Chronic alcohol consumption can lead to immunosuppression, impairing the body's ability to respond to infection and tissue injury (5). Alcohol-mediated immune dysfunction may alter cytokine signaling, reduce antimicrobial defenses, and compromise tissue repair, thereby increasing susceptibility to infection and organ damage. Chronic alcohol consumption may impair interferon-mediated antiviral signaling and enhance hepatitis C virus (HCV) replication, leading to potentiated HCV-associated liver injury (6–8). Of note, an *in vivo* study by Sepúlveda et al. has shown that chronic alcohol use aggravates coxsackievirus-induced myocarditis by suppressing the function of type 1 helper T (Th1) cells in a murine acquired immunodeficiency syndrome model (9). However, the molecular mechanism underlying alcohol-induced immunosuppression in the heart remains unclear.

Dysregulation of circadian clock genes contributes to different types of cardiovascular disease (CVD) (10). The cardiomyocyte circadian clock regulates energy metabolism, calcium homeostasis, electrical activity, cardiomyocyte contraction, and insulin sensitivity (11). In particular, disruption of circadian signaling can impair cardiomyocyte electrical and contractile functions and increase susceptibility to arrhythmias and cardiac dysfunction (11). For example, disrupting the clock genes, key circadian regulators, promotes aging-related cardiomyopathy (12). However, the role of chronic alcohol use in affecting circadian gene regulation in the myocardium has not been explored.

In our previous study using rats fed a Lieber-DeCarli ethanol diet, we demonstrated that chronic ethanol exposure induced myocardial oxidative stress and mitochondrial dysfunction (13). However, a comprehensive assessment of myocardial transcriptional responses to chronic ethanol exposure in the left ventricle remains limited. Therefore, the objective of the present study is to characterize ethanol-induced alterations in the left ventricular transcriptome and identify molecular pathways potentially contributing to chronic alcohol-associated cardiac injury by using bulk RNA sequencing and Ingenuity Pathway Analysis (IPA). Our findings identified detrimental alterations in myocardial gene expression mainly related to immunosuppression and circadian rhythm, providing new insight into the molecular effects of chronic alcohol exposure in the heart.

## 2 Method

### 2.1 Study design

Male Wistar rats weighing 175–200 g were obtained from Charles River Laboratories (Portage, MI, USA). Rats were maintained under a standard 12:12-hour light-dark cycle. Rats were weight-matched and randomly assigned to either an ethanol-fed group (ET; *n* = 5) or an isocaloric control group (CONT; *n* = 4). ET rats received a Lieber-DeCarli liquid diet containing 6.7% (v/v) ethanol (Dyets Inc., Bethlehem, PA, USA), while CONT rats received an isocaloric liquid control diet matched to the amount consumed by the ethanol-fed rats, thereby minimizing differences in caloric intake between groups. Following a 6-day ethanol ramp-up period from 0% to 6.7%, rats were maintained on their respective diets for 6 weeks, as previously described (13). This model is a well-established model used to study the effects of chronic alcohol exposure on cardiac injuries in rodents (13–15). At the end of the study, rats were deeply anesthetized with 5% isoflurane. The thoracic cavity was opened, and blood and hearts were collected. Euthanasia was completed by exsanguination and bilateral pneumothorax, and tissues were processed as appropriate for subsequent analyses. Left ventricular tissues were rapidly excised, snap-frozen in liquid nitrogen, and stored at −70 °C until analysis. The left ventricle was collected for further analysis because it is the main pumping chamber of the heart, and its dysfunction is a major feature of cardiomyopathy and heart failure (16). Thus, analysis of left ventricular tissue provides a relevant approach for identifying myocardial molecular alterations associated with ACM. The animal protocol was approved by the Institutional Animal Care and Use Committee of the VA Nebraska-Western Iowa Healthcare System Research Service (#1616192; approved April 2021).

### 2.2 RNA sequencing

Approximately 35 mg of left ventricular tissue was homogenized in TRIzol reagent, and total RNA was isolated according to the manufacturer's instructions. RNA quality and concentration were assessed using NanoDrop Lite Plus Spectrophotometers (Thermo Fisher Scientific, MA, USA). RNA integrity was assessed before library preparation. RNA-sequencing libraries were generated from 500 ng of total RNA per sample using the NuGEN Universal Plus RNA-Seq Library Preparation Kit according to the manufacturer's instructions. Sequencing was performed using an Illumina NovaSeq 6000 system. The raw counts were used for differentially expressed gene analysis by DESeq2. FPKM values were used only for expression visualization.

Genes with an Benjamini-Hochberg adjusted *P* value < 0.05 were considered differentially expressed.

### 2.3 Ingenuity pathway analysis (IPA)

Differentially expressed genes were subsequently analyzed using IPA (QIAGEN, Netherlands) to identify canonical pathways altered in the left ventricle following chronic ethanol exposure. The processed significantly altered gene expression dataset was uploaded to IPA and analyzed using the Ingenuity Knowledge Base (genes and endogenous chemicals) as the reference set. Both direct and indirect relationships were included, along with endogenous chemicals. Canonical pathways and related visualizations were generated using QIAGEN IPA Interpret, with a false discovery rate-adjusted *P* value < 0.05 considered statistically significant.

### 2.4 Real-time PCR

The total RNA from rat left ventricles was reverse-transcribed utilizing iScript Supermix (1708841, Bio-Rad, CA, USA). Real-time PCR was further performed to measure the transcript levels of glyceraldehyde-3-phosphate dehydrogenase (*Gapdh*), interferon-induced protein with tetratricopeptide repeats 3 (*Ifit3*), interferon regulatory factor 9 (*Irf9*), basic helix-loop-helix ARNT like 1 (*Bmal1*), D-box binding PAR BZIP transcription factor (*Dbp*), Patatin like domain 3, 1-acylglycerol-3-phosphate O-acyltransferase (*Pnpla3*), and selectin *P* (*Selp*). The TaqMan™ real-time PCR primer probes from Applied Biosystems (MA, USA) were used to detect the mRNA levels of genes, including *Ifit3* (Rn01479928_m1), *Irf9* (Rn01489163_m1), *Bmal1* (Rn00577590_m1), *Dbp* (Rn01498425_m1), *Pnpla3* (Rn01502361_m1) and *Selp* (Rn00565416_m1). Transcript levels were normalized to *Gapdh* (Rn01775763_g1). A *ΔΔ*CT method was utilized for the quantification.

### 2.5 Statistical analysis

For RNA-seq analysis, the Benjamini-Hochberg (B-H) method was used to adjust the *P* value for multiple-testing-caused false discovery rate. Adjusted *P* < 0.05 was considered significant. For real-time PCR, single-factor comparisons were performed by two-tailed Student's t-test. All data are shown as mean ± the standard error of the mean (SEM). Differences at *P* < 0.05 were considered statistically significant. GraphPad Prism 10.1 was used for the statistical analysis.

## 3 Results

### 3.1 Chronic ethanol feeding alters the left ventricular transcriptome

To determine the effects of chronic ethanol consumption on myocardial gene expression, bulk RNA sequencing was performed using left ventricular tissues from ethanol-fed and isocaloric control-fed rats. Of all differentially expressed genes, 393 were significantly altered, including 194 upregulated and 199 downregulated genes (Adjusted *P* < 0.05, Figure 1A). The significantly altered genes were subsequently analyzed by IPA to detect altered signaling pathways.

**Figure 1:**
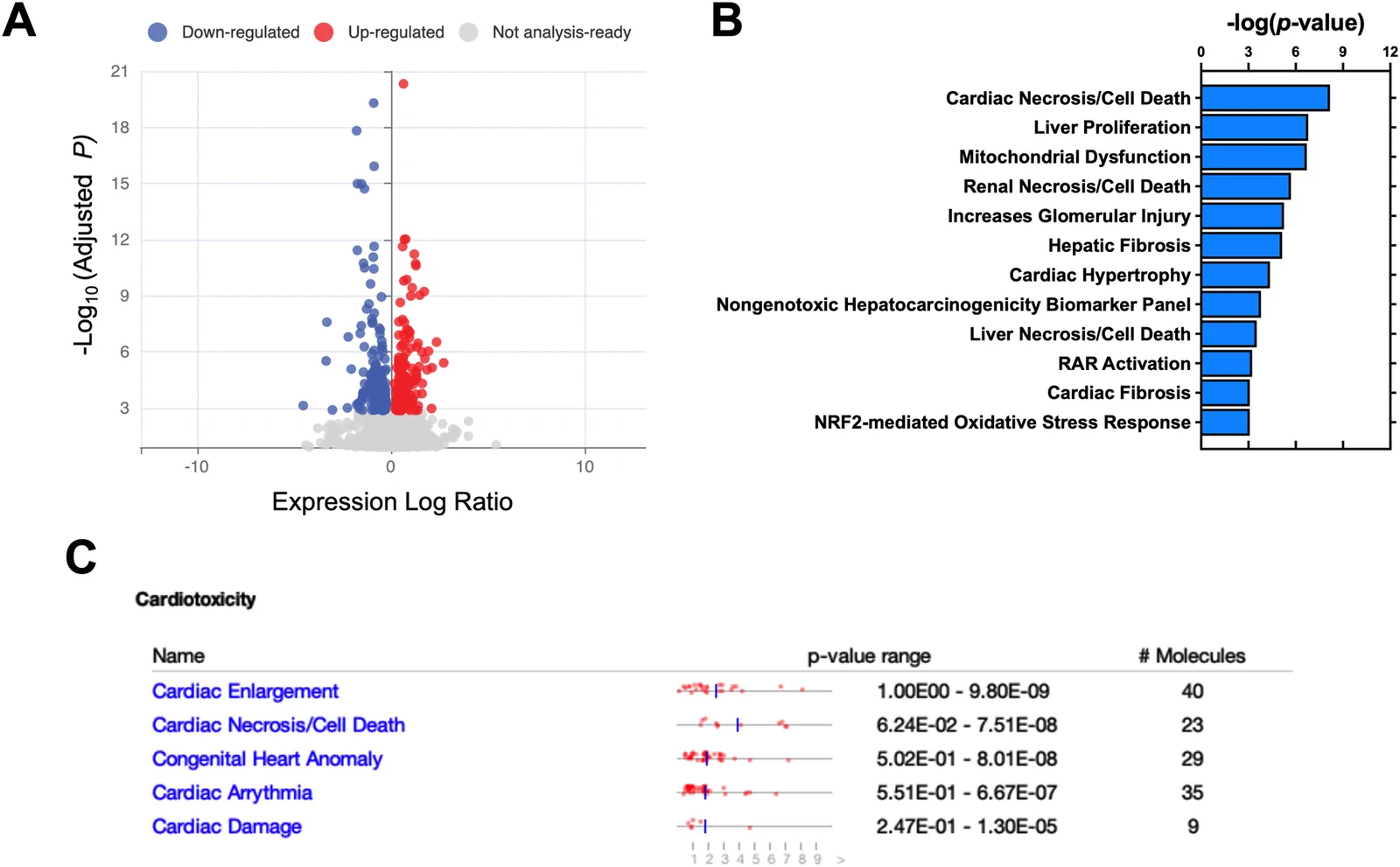
Identification of significantly altered genes associated with ACM. **(A)** Volcano plots showing differentially expressed genes (adjusted *P* < 0.05) in the hearts of ET-fed rats (*n* = 5) compared with CONT-fed rats (*n* = 4). Blue reflects downregulated genes, and red reflects upregulated genes. **(B)** Tox Lists analysis shows the top 12 organ lesions altered in the myocardium of ET-fed rats. **(C)** Tox Functions analysis identifies cardiotoxicity-related outcomes associated with the altered cardiac transcriptome of ET-fed rats. Data were analyzed using QIAGEN IPA (QIAGEN Inc., https://digitalinsights.qiagen.com/IPA).

Toxicity (Tox) lists and Tox Functions detected by IPA can link molecular and experimental observations to clinically relevant toxicity features. This enables deeper investigation into disease-induced biological effects and helps uncover the pathways and mechanisms responsible for both therapeutic benefits and adverse toxicological outcomes. Tox Lists analysis (Figure 1B) shows that myocardial genes from ET-fed rats were significantly associated with cardiac necrosis/cell death (*P* = 6.79 × 10^−9^), liver proliferation (*P* = 1.61 × 10^−7^), and mitochondrial dysfunction (*P* = 2.02 × 10^−7^). In addition, Tox Functions analysis (Figure 1C) identifies several cardiotoxicity-related outcomes associated with the altered cardiac transcriptome of ET-fed rats. The top five cardiotoxic features include cardiac enlargement, cardiac necrosis/cell death, congenital heart anomaly, cardiac arrhythmia, and cardiac damage. In particular, the cardiac enlargement and cardiac arrhythmia categories include 40 and 30 significantly altered genes identified in the present study, respectively. These data support the notion that ethanol-induced alterations in gene expression may contribute to maladaptive cardiac remodeling and increased susceptibility to cardiac arrhythmias.

### 3.2 Immune and circadian rhythm pathways are downregulated by chronic ethanol feeding

To identify the biological pathways represented by the significantly altered genes, canonical pathway analysis was performed using IPA, as shown in Figure 2A. Among the top 25 altered pathways ranked by Q value, IPA identified several downregulated pathways involved in immune regulation and related signal transduction. Interferon-α/*β* signaling was significantly downregulated in the left ventricles of ethanol-fed rats compared with control-fed rats (*P* = 7.76 × 10^−8^, *z*-score = −2.53). Consistent with this pathway-level prediction, multiple genes involved in interferon-α/*β* signaling, including *Eif2ak2, Gbp2, Ifit2, Ifit3, Irf6, Irf9, Oasl, Stat2, and Usp18* (Table 1), were downregulated in the myocardium of ET-fed rats. Of note, signal transducer and activator of transcription 2 (STAT2), a central component of type I and type III interferon signaling, was downregulated following chronic ethanol feeding. This finding was accompanied by a predicted inhibition of host responses by IFN-stimulated genes (*P* = 7.41 × 10^−6^, *z*-score = −1.342), further supporting the suppression of myocardial interferon-responsive signaling. Given the critical role of interferons in the host defense process (17), inhibited Interferon-α/*β* signaling by chronic alcohol exposure indicates immunosuppression in the heart. Consistent with the adverse alteration in interferon-α/*β* signaling, pathways involved in pattern recognition receptor-mediated recognition of bacteria and viruses, as well as interferon-*γ* signaling, were also downregulated following chronic ethanol exposure.

**Figure 2:**
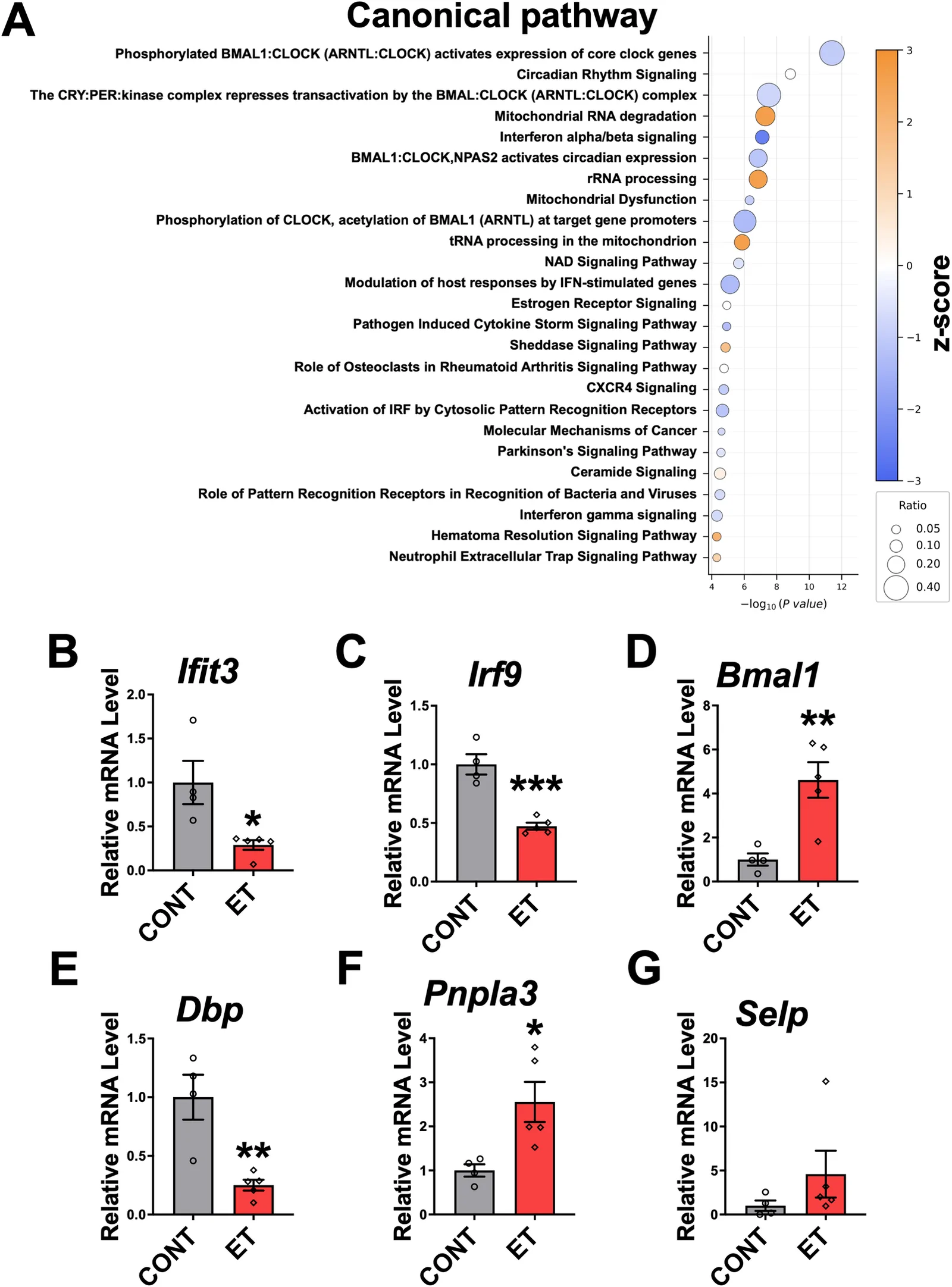
Canonical pathway analysis of the significantly altered genes associated with ACM. **(A)** The top 25 most significantly altered canonical pathways in the myocardium of ET-fed rats versus CONT-fed mice were listed. Yellow color (positive z-score) indicates upregulation of pathways, and blue color (negative z-score) stands for downregulation. **(B–G)** Real-time PCR analysis showing transcript levels for *Ifit3*, *Irf9*, *Bmal1*, *Dbp*, *Pnpla3*, and *Selp* in myocardium of ET (*n* = 5) and CONT (*n* = 4) rats.

**Table 1:** Selected genes and pathways altered in myocardium by chronic ethanol exposure.

Gene Name	Expr Log Ratio	Q value	Location	Type
Interferon-α/*β* signaling				
*Eif2ak2*	−0.628	0.000513	Cytoplasm	kinase
*Gbp2*	−0.685	0.000844	Cytoplasm	other
*Ifit2*	−0.824	7.49E-06	Cytoplasm	other
*Ifit3*	−1.48	0.000417	Cytoplasm	other
*Irf6*	−0.818	0.000977	Nucleus	transcription regulator
*Irf9*	−0.769	6.86E-06	Nucleus	transcription regulator
*Oasl*	−1.227	0.0001	Cytoplasm	enzyme
*Stat2*	−0.47	0.00131	Nucleus	transcription regulator
*Ubc*	0.276	0.000396	Cytoplasm	enzyme
*Usp18*	−0.768	0.00122	Cytoplasm	peptidase
Phosphorylated BMAL1:CLOCK (ARNTL:CLOCK) activates expression of core clock genes				
*Bhlhe40*	−0.869	3.06E-09	Nucleus	transcription regulator
*Bmal1*	2.117	0.00111	Nucleus	transcription regulator
*Cry2*	−0.571	3.62E-05	Nucleus	enzyme
*Dbp*	−2.046	0.00126	Nucleus	transcription regulator
*Npas2*	1.398	9.37E-05	Nucleus	transcription regulator
*Per1*	−1.416	1.67E-08	Nucleus	transcription regulator
*Per2*	−1.728	2.39E-12	Nucleus	transcription regulator
*Per3*	−1.769	7.89E-15	Nucleus	other
*Sik1*	−0.991	0.0126	Cytoplasm	kinase
Mitochondrial RNA degradation/tRNA processing in the mitochondrion				
*Mt-co1*	0.452	0.0248	Cytoplasm	kinase
*Mt-co3*	0.458	0.0358	Cytoplasm	other
*Mt-nd1*	0.76	0.00116	Cytoplasm	other
*Mt-nd2*	0.591	0.0211	Cytoplasm	other
*Mt-nd4*	0.67	0.0402	Nucleus	transcription regulator
*Mt-nd5*	0.575	0.00109	Nucleus	transcription regulator
*Mt-nd4l*	0.509	0.00792	Cytoplasm	enzyme
Sheddase signaling pathway				
*Adam17*	0.497	0.00251	Plasma Membrane	peptidase
*Ccrl2*	−0.402	0.0454	Plasma Membrane	G-protein coupled receptor
*Col4a3*	−0.432	0.0487	Extracellular Space	other
*Fos*	−1.418	0.00162	Nucleus	transcription regulator
*Igf1*	0.621	0.00214	Extracellular Space	growth factor
*Igfbp4*	0.792	0.000227	Extracellular Space	other
*Mmp14*	0.545	0.0161	Extracellular Space	peptidase
*Mmp17*	0.642	0.00491	Extracellular Space	peptidase
*Pik3c2a*	−0.589	0.0143	Cytoplasm	kinase
*Pik3c2g*	2.351	8.15E-05	Cytoplasm	kinase
*Selp*	1.608	0.000223	Plasma Membrane	transmembrane receptor
*Zbtb16*	−1.448	0.0115	Nucleus	transcription regulator
Neutrophil extracellular trap signaling pathway				
*Clec7a*	1.103	0.0238	Plasma Membrane	transmembrane receptor
*Col5a3*	0.822	0.0153	Extracellular Space	other
*Col8a2*	0.673	0.0282	Extracellular Space	other
*Itpr3*	−0.344	0.0364	Cytoplasm	ion channel
*Nox4*	−1.006	0.0312	Cytoplasm	enzyme
*Pnpla3*	1.486	5.39E-07	Cytoplasm	enzyme
*Prkcd*	0.57	0.004	Cytoplasm	kinase
*Prkcq*	−0.707	0.0141	Cytoplasm	kinase
*Selp*	1.608	0.000223	Plasma Membrane	transmembrane receptor

Additionally, the IPA identified altered circadian rhythm regulation in the left ventricles of ethanol-fed rats (Figure 2A). For example, the molecular pathways related to circadian rhythm regulation, including phosphorylated BMAL1:CLOCK (ARNTL:CLOCK), which activates the expression of core clock genes (*P* = 3.98 × 10^−12^, *z*-score = −1), and the CRY:PER:kinase complex, which represses transactivation by the BMAL:CLOCK (ARNTL:CLOCK) complex (*P* = 3.02 × 10^−8^, *z*-score = −0.816), are both downregulated in the rat myocardium upon ET feeding. Regarding core molecular regulators of the circadian clock, we noted that myocardial genes related to CRY:PER:kinase complex, including *Cry2, Per1, Per2,* and *Per3* (Table 1)*,* are significantly downregulated in the myocardium of ET-fed rats. Additionally, *Dbp*, a key clock-controlled gene, was downregulated in myocardium of ET-fed rats (Table 1). Of note, genes of CLOCK:BMAL1 complex, such as *Bmal1* and neuronal PAS domain protein 2 *(Npas2,* Table 1)*,* were significantly upregulated in ET-fed rats compared with CONT-fed rats. BMAL1 and NPAS2 form a transcriptional complex that regulates circadian gene expression and participates in cardiac metabolic and stress responses (18). Therefore, altered expression of these genes further supports dysregulation of the myocardial circadian transcriptional network following chronic ethanol exposure, which may contribute to cardiac dysfunction.

We next performed quantitative real-time PCR to validate representative genes from the major pathways identified by RNA-seq. Among the interferon-related genes, *Ifit3* (*P* < 0.05, Figure 2B) and *Irf9* (*P* < 0.001, Figure 2C) were significantly downregulated in the myocardium of ethanol-fed rats compared with CONT rats. For genes mediating circadian clock regulation, *Bmal1* (*P* < 0.01, Figure 2D) was significantly upregulated, whereas *Dbp* was significantly decreased (*P* < 0.01, Figure 2E) in the myocardium of ET-fed rats. These results further support chronic ethanol exposure-induced dysregulation of interferon signaling and circadian rhythm.

### 3.3 Pathways upregulated in the left ventricle following chronic ethanol feeding

Among the top 25 altered pathways, pathways related to mitochondrial RNA degradation (*P* = 5.01 × 10^−8^, z score = 2.646), tRNA processing in the mitochondrion (*P* = 1.35 × 10^−6^, z score = 2.646), sheddase signaling (*P* = 1.41 × 10^−5^, z-score = 1.732), and neutrophil extracellular trap signaling (*P* = 4.90 × 10^−5^, z-score = 1.213) are significantly upregulated in the left ventricles of ethanol-fed rats compared with control-fed rats (Figure 2A).

Chronic alcohol exposure has been shown to induce mitochondrial dysfunction in the myocardium (1). The enrichment of mitochondrial RNA degradation or tRNA processing-related genes, including *Mt-co1*, *Mt-co3*, *Mt-nd1*, *Mt-nd2*, *Mt-nd4*, *Mt-nd4l*, Mt-*nd5* (Table 1), suggests that chronic ethanol exposure alters pathways involved in mitochondrial RNA processing or turnover. These findings identify dysregulated mitochondrial RNA processing as a potential mechanism associated with myocardial mitochondrial dysfunction. Chronic ethanol feeding also upregulated several genes associated with sheddase signaling, including *Adam17, Igf1, Igfbp4, Mmp14, Mmp17,* and *Selp* (Table 1), indicating altered proteolytic processing of membrane-bound and extracellular matrix (ECM) proteins. In addition, genes associated with neutrophil extracellular traps (NETs), including *Clec7a, Col5a3, Col8a2, Pik3c2g, Pnpla3, Prkcd, and Selp,* are also upregulated, indicating an increase in neutrophil-associated inflammation in the myocardium caused by chronic alcohol exposure. In addition, among the NET-related genes, ethanol-fed rats showed an increased gene expression of *Pnpla3* (*P* < 0.05, Figure 2F) and a trend toward an increase in *Selp* expression (*P* = 0.28, Figure 2G) in the myocardium compared with CONT rats.

Collectively, IPA indicates that chronic ethanol consumption suppresses interferon response pathways and disrupts circadian rhythm regulation, accompanied by changes in gene expression associated with impaired mitochondrial RNA homeostasis, increased sheddase-mediated signaling, and enhanced neutrophil-mediated inflammation in rat myocardium.

### 3.4 Upstream analysis identifies the top regulator mediating the adverse effects of ethanol on rat ventricle

We next performed IPA upstream analysis to identify cytokines and chemical compounds that may regulate the myocardial transcriptional changes induced by chronic ethanol exposure. Figure 3A presents the top 7 predicted upstream regulators, ranked according to their *P* value of overlap. In particular, Interferon-*γ* (IFNG) was identified as the highest-ranked upstream regulator and was predicted to be significantly inhibited in the left ventricles of ethanol-fed rats (*P* = 2.85 × 10^−37^, *z*-score = −3.042. IFNG was predicted to regulate 130 differentially expressed genes identified in the present study. In addition, Interferon-*α* (IFNA) was identified as another upstream regulator (*P* = 4.90 × 10^−20^, *z*-score = −3.124) and was predicted to regulate 44 differentially expressed myocardial genes in ET-fed rats.

**Figure 3:**
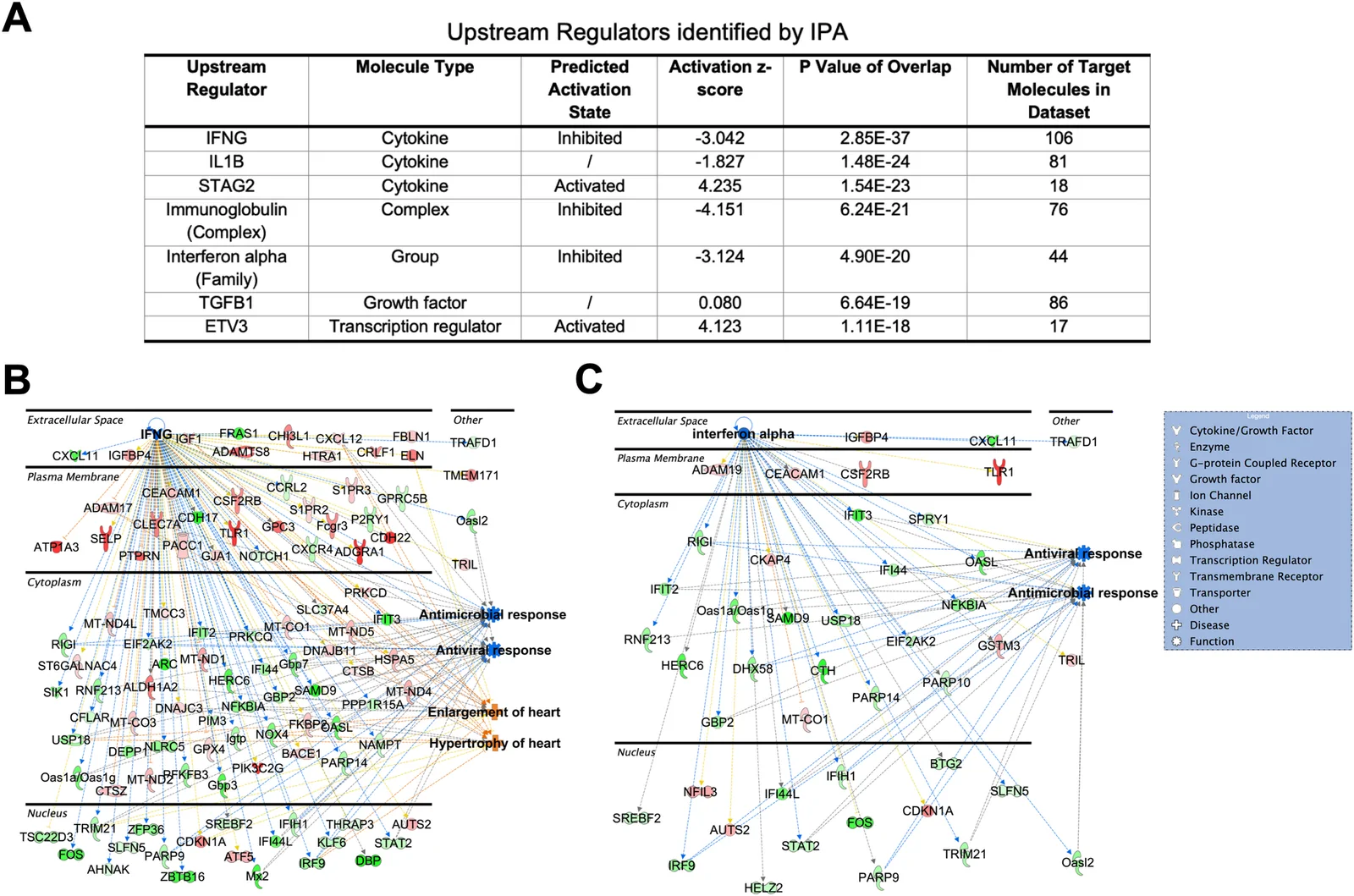
Upstream regulator analysis of the significantly altered myocardial genes in ET-fed rats. **(A)** Top 7 Upstream regulators identified by IPA; **(B)** IFNG and overlapped genes predicted to be regulated by IFNG in myocardium following alcohol exposure are associated with suppression of antimicrobial and anti-viral responses and activation of hypertrophy of the heart; **(C)** IFNA-regulated overlapped genes in rat myocardium upon alcohol exposure are associated with inhibition of antimicrobial and anti-viral responses. Data were analyzed using QIAGEN IPA (QIAGEN Inc., https://digitalinsights.qiagen.com/IPA).

Using the IPA “Grow” function, we further identified Diseases and Functions annotations associated with the IFNG*-*regulated genes (Figure 3B). These genes were linked to predicted activation of cardiac hypertrophy (B-H correction-adjusted *P* = 2.24 × 10^−7^, *z*-score = 1.427) and predicted inhibition of antiviral responses (B-H correction-adjusted *P* = 6.45 × 10^−21^, *z*-score = −2.388, overlapping 21 genes) and antimicrobial response (B-H correction-adjusted *P* = 1.23 × 10^−21^, *z*-score = −2.386, overlapping 25 genes). Additionally, we noted that IFNA-regulated genes (Figure 3C) were tightly linked to signaling pathways related to antiviral responses (B-H correction-adjusted *P* = 1.78 × 10^−23^, *z*-score = −1.989, overlapping 18 genes) and antimicrobial response (B-H correction-adjusted *P* = 1.44 × 10^−21^, *z*-score = −1.989, overlapping 19 genes). Collectively, these findings associate predicted inhibition of IFNG and IFNA signaling in the ethanol-exposed myocardium with suppression of antiviral and antimicrobial gene-expression programs and a transcriptional profile related to cardiac hypertrophy.

## 4 Discussion

The present study characterized the left ventricular transcriptome in response to chronic ethanol exposure in male Wistar rats. Chronic ethanol feeding altered 393 myocardial genes, including 194 upregulated and 199 downregulated genes. Pathway analysis identified a coordinated transcriptional profile indicating suppressed interferon-mediated host-defense pathways, and disrupted myocardial circadian rhythm regulation. Further, we noted an upregulation of pathways involved in mitochondrial RNA processing, sheddase signaling, and neutrophil-associated inflammation. In addition, upstream regulator analysis identified IFNG and IFNA as the highest-ranking predicted upstream regulators. The ethanol-induced inhibition of the interferon-regulated gene network was associated with downregulation of genes involved in antiviral and antimicrobial responses and upregulation of genes related to cardiac hypertrophy.

A major finding of the present study is the predicted inhibition of interferon-related signaling, which is characterized by downregulated myocardial genes, including *Eif2ak2, Gbp2, Ifit2, Ifit3, Irf6, Irf9, Oasl, Stat2,* and *Usp18,* in ET-fed rats. Among these genes, *Stat2* and *Irf9* are central components of interferon signal transduction, whereas *Eif2ak2, Ifit2, Ifit3,* and *Oasl* participate in downstream antiviral responses. Specifically, STAT2 binds to the IRF-association domain of IRF9 (19), which plays an important role in regulating interferon-responsive transcription (20). Similarly, eukaryotic translation initiation factor 2 alpha kinase 2 (EIF2AK2), an interferon-stimulated enzyme, inhibits viral replication through phosphorylation of eIF2*α* (21). In addition, interferon-induced protein with tetratricopeptide repeats 2 (IFIT2) and IFIT3 can form a complex mediating the restriction of viral RNA translation. A recent study from Glasner et al. has proved the antiviral role of the IFIT2-IFIT3 complex by targeting short 5' untranslated regions of viral mRNAs (22). Consistent with this interpretation, pathways involving PKR-mediated interferon induction and antiviral responses, DDX58/IFIH1-mediated induction of interferon-α/*β*, and modulation of host responses by interferon-stimulated genes were also predicted to be inhibited. These findings suggest that chronic ethanol exposure possibly suppresses myocardial antiviral immune signaling, potentially increasing susceptibility to viral infection and virus-induced myocardial injury. Of note, a comparative study using an immunocompromised mouse model has shown that ethanol consumption leads to a higher incidence of coxsackievirus-induced myocarditis and more severe virus-induced cardiac lesions (9). These adverse effects are mediated by ethanol-induced cytokine imbalance favoring a Th2 response and suppression of Th1 function. Although this study provided novel evidence linking ethanol consumption to more severe virus-induced cardiac injury, the underlying molecular mechanisms remain to be further defined. In the present study, the upstream regulator analysis further strengthened the role of interferon-related signaling by identifying IFNG and IFNA as the significant upstream regulators. The IFNG-regulated network was also associated with predicted activation of cardiac hypertrophy and predicted inhibition of antiviral response. The present findings identify impaired myocardial interferon signaling as a potential molecular link among chronic alcohol exposure, reduced antiviral immunity, and myocardial remodeling, providing a basis for future mechanistic investigation.

Circadian dysregulation represents another prominent feature of the ethanol-exposed myocardium. Pathways involving phosphorylation and nuclear translocation of the CRY:PER:kinase complex, CLOCK phosphorylation, and BMAL1 acetylation at target-gene promoters were predicted to be inhibited. Of note, myocardial genes related to CLOCK:BMAL1 complex, including *Bmal1* and *Npas2,* were significantly increased in response to ET exposure. BMAL1 forms a heterodimer with NPAS2 that functions as a redox sensor and plays an important role in maintaining metabolic homeostasis (23). In terms of CVD, myocardial infarction has been shown to enhance the expression of *Bmal1* and *Npas2,* in myocardium, along with increased occurrence of ventricular tachyarrhythmias in rats (24). Cardiomyocyte BMAL1 has been linked to hypertrophy, oxidative stress, apoptosis, and fibrotic signaling (25, 26). In contrast to the increased expression of *Bmal1* and *Npas2*, myocardial genes encoding the CRY:PER:kinase complex, including *Cry2, Per1, Per2, and Per3*, were downregulated following chronic ethanol exposure. Period circadian regulator (PER) and cryptochrome circadian regulator (CRY) proteins normally form a repressor complex that inhibits BMAL1-containing transcriptional complexes and maintains circadian rhythm (27). Therefore, the opposing expression patterns of the positive and negative arms of the molecular clock suggest disruption of the myocardial circadian transcriptional feedback network rather than a uniform increase or decrease in clock activity. Among these genes, *Per2* is particularly relevant to myocardial stress response. Loss of *Per2* enhances tumor necrosis factor-α- or H_2_O_2_-induced mitochondrial dysfunction and apoptosis in cardiomyocytes (28), suggesting that reduced *Per2* expression may impair mitochondrial resilience under inflammatory and oxidative stress. Collectively, these findings identify the myocardial circadian clock as a previously underrecognized target of chronic ethanol exposure. The abnormal upregulation of *Bmal1* and *Npas2* and downregulation of *Cry2* and *Per1–3* suggest disruption of circadian feedback regulation, potentially linking ethanol exposure to mitochondrial dysfunction, oxidative stress, and myocardial remodeling. However, because gene expression was assessed at a single time point in the present study, future studies examining cardiac clock-related gene expression across the light-dark cycle will help determine whether circadian clock disruption contributes to ethanol-induced cardiac dysfunction and remodeling.

Mitochondrial RNA degradation was one of the important pathways enriched in the ethanol-exposed myocardium. This pathway included several mitochondria-encoded genes, such as *Mt-co1, Mt-co3, Mt-nd1, Mt-nd2, Mt-nd4, Mt-nd4l,* and *Mt-nd5*, which encode essential components of respiratory-chain complexes I and IV. Given that mitochondrial RNA processing and turnover are essential for the expression of oxidative phosphorylation components, disruption of these processes may impair mitochondrial respiration and myocardial energy production (29). This interpretation is consistent with previous studies demonstrating that chronic ethanol exposure decreases total RNA and mitochondrial ribosomal RNA in rat left ventricles (30). Of note, Zhang et al. have shown that ethanol can destabilize fas-activated serine/threonine kinase (FASTK), a key modulator for mitochondrial RNA processing by interacting with mitochondrial RNA granules (31). In this study, deletion of *Fastk* decreases *Mt-nd6* gene expression, which promotes ethanol-induced decreases in mitochondrial complex I gene expression and impairs myocardial mitochondrial function in mouse ACM model. Related to these observations, we recently have reported that ethanol decreased the protein level of NADH:ubiquinone oxidoreductase subunit B8 (NDUFB8), a mitochondrial complex I protein, which was associated with mitochondrial dysfunction (13). Together, the enrichment of mitochondrial RNA degradation pathways associated with mitochondrial complex I dysfunction in ACM supports dysregulation of mitochondrial RNA homeostasis as an important molecular feature of the ethanol-exposed myocardium.

Chronic ethanol feeding also increased the expression of genes associated with sheddase signaling, including *Adam17, Igf1, Igfbp4, Mmp14, Mmp17,* and *Selp*. Sheddases regulate intercellular communication by proteolytically releasing membrane-bound cytokines, receptors, growth factors, and adhesion molecules (32). In particular, a disintegrin and metalloproteinase domain 17 (ADAM17) cleaves numerous membrane-associated substrates involved in myocardial inflammation and remodeling in heart failure (33). For example, cardiomyocyte-specific ADAM17 deletion attenuates doxorubicin-induced myocardial remodeling and dysfunction (34). In addition, matrix metallopeptidase 14 (MMP14) is a membrane-associated metalloproteinase involved in ECM processing and cardiac injury responses (35). Several lines of studies have demonstrated the role of MMP14 in vascular remodeling, atherosclerosis, cardiac repair, and heart failure-related remodeling. Specifically, a recent finding from Plavelil et al. has shown that collagen deposition is increased in a heart failure swine model, which is associated with an increase in myocardial MMP14 (36). In a separate study, Yu et al., using a transverse aortic constriction-induced heart failure mouse model, have reported that a decrease in MMP14 expression by miR-150–5p attenuates myocardial hypertrophy, fibrosis, and systolic dysfunction in heart failure (37). Together, these findings identify sheddase signaling, particularly the ADAM17- and MMP14-associated proteolytic networks, as a critical altered pathway in response to chronic ethanol exposure. This pathway provides new mechanistic insight into alcohol-associated myocardial remodeling and highlights sheddases as promising candidates for future functional investigation.

Another notable finding was the upregulation of genes related to NET signaling, including *Clec7a, Col5a3, Col8a2, Pik3c2g, Pnpla3, Prkcd, and Selp*. NETs are extracellular structures containing DNA, histones, and antimicrobial proteins that are released by activated neutrophils (38). In particular, excessive NET accumulation has been implicated in cardiovascular inflammation, thrombosis, myocardial injury, and tissue remodeling. In a clinical study, increased NET formation is found in the hearts of dilated cardiomyopathy patients (39). An increased number of NETs in the myocardium is associated with decreased left ventricular ejection fraction and higher B-type natriuretic peptide, a heart failure marker. Additionally, *Selp*, a gene encoding a cell adhesion molecule, mediates the initial tethering and recruitment of circulating neutrophils to the endothelial border during organ injury (40). In an isolated rat heart model of ischemia-reperfusion, *P*-selectin inhibition reduced neutrophil-mediated myocardial injury and improved left ventricular function and coronary flow (41). Consequently, this transcriptional profile should be interpreted as a NET-associated pathway signature, rather than direct evidence that NET formation occurred in the myocardium. Therefore, the enrichment of NET signaling identify a potentially important connection among chronic ethanol exposure, endothelial activation, neutrophil recruitment, and myocardial remodeling.

Several limitations warrant consideration. The exclusive use of male rats limits the broader applicability of these findings, given established sex-dependent differences in responses to chronic alcohol exposure. Because bulk RNA-seq was performed to observe the global gene changes in left ventricular tissue, the cell type-specific changes could not be resolved, and contributions from different cardiac cells, including cardiomyocytes, fibroblasts, endothelial cells, vascular cells, and immune cells, remain to be defined. While the present study identified upregulation of NET-associated genes, these transcriptional changes do not directly establish increased neutrophil infiltration or NET formation in the myocardium. Future studies quantifying infiltrated myocardial neutrophil counts and established NET markers will be required to validate the biological and functional significance of NET formation-related pathways in ACM.

Together, these findings expand the current understanding of ACM by identifying alterations in core circadian clock pathways and interferon-mediated immune responses that may be associated with mitochondrial RNA processing, sheddase-mediated signaling, and NET-related inflammatory responses in the ethanol-exposed myocardium. Further cell-specific and functional studies are needed to determine how these pathways interact and whether restoration of interferon or circadian signaling can limit the development of ACM.

## Data Availability

The datasets presented in this study can be found in online repositories. The names of the repository/repositories and accession number(s) can be found below: https://www.ncbi.nlm.nih.gov/geo/, GSE343812.
